# Simple Technique for Augmentation of the Facial Soft Tissue

**DOI:** 10.1100/2012/262989

**Published:** 2012-04-26

**Authors:** Francesco Inchingolo, Marco Tatullo, Fabio M. Abenavoli, Massimo Marrelli, Alessio D. Inchingolo, Roberto Corelli, Raffaella Mingrone, Angelo M. Inchingolo, Gianna Dipalma

**Affiliations:** ^1^Department of Dental Sciences and Surgery, University of Bari, 70121 Bari, Italy; ^2^Department of Maxillofacial Surgery, Calabrodental, 88900 Crotone, Italy; ^3^Department of Basic Medical Sciences, University of Bari, 70121 Bari, Italy; ^4^Department of “Head and Neck Diseases”, Hospital “Fatebenefratelli”, 00186 Rome, Italy; ^5^Department of Maxillofacial Surgery, University of Bari, 70121 Bari, Italy; ^6^Department of Surgical, Reconstructive and Diagnostic Sciences, University of Milano, 20122 Milano, Italy

## Abstract

Due to the request of numerous patients to improve the aspect of the perioral area in combination with other types of cosmetic and reconstructive surgery, we started to use autologous fillers. In fact, there are numerous potential fillers that can be utilized during various operations executed in many bodily areas, such as the breast, abdomen, and face. The muscular fascia as well as the dense connective tissue which the surgeon encounters in various bodily areas during some stages of the operation, in fact, can be removed and replaced both by themselves or superimposed in order to increase their thickness. The insertion of the grafts is carried out by using a needle, but other methods can also be used with the same success. The consistency of the area treated, after a few days of edema, is very similar to the host area, and the volume obtained remains uniform in time (our followup is after 24 months). The time utilized for the removal and the insertion in the chosen area was only a few minutes. The result was extremely satisfactory in all the 30 patients treated, and there was no complication or side effects.

## 1. Introduction

The use of fillers to improve the aesthetic aspect of the face is now a frequently used methodology. Numerous products have been proposed and utilized with variable success. Generally, what is required of these products is their biocompatibility, stability, naturalness, and reproducibility of the result obtained. Another important factor is that the consistency of the area treated with these fillers is such that at the end of the filling there are no differences of consistency between the surrounding areas. Certainly, the fact that these products are easily available, utilizable, and having a minimum of side effects is also very important. However, since there is a good deal of research for new products going on, this seems to be indicative of the fact that the objectives proposed have not yet been reached.

 Two of the most interesting areas for the application of various products are those of the nasal-labial folds and the lips. In fact, in these areas, the passing of time as well as in many cases the bad habit of smoking, dental prosthesis that are not perfect and other elements directly harm their aesthetic aspect. In any case, the presence of well-defined turgid lips is the guarantee of aesthetic attractiveness regardless of one's age. This desire, therefore, explains why there are so many requests to correct these areas, both as a single procedure or in association with other plastic surgery operations.

 The tissue utilized must guarantee an adequate consistency, so that it can be easily shaped and inserted in the area. Tissues which certainly have these characteristics are that of the muscular fascia so as the most compact tissue that is found, in varying amounts, in the subcutaneous thickness of all bodily regions. Therefore, with this principle in mind and considering how it was possible to find suitable autologous tissue, we began to select and utilize this material in different operations.

## 2. Materials and Methods

After having evaluated the requests and expectations of the patients, also in relation to the type of operation programmed, we started to graft the muscular fascia which had been removed during some operations. In the case of mastoplasty operations [1], both of augmentation and reduction, the pectoral muscle fascia was easily accessible for a large tract allowing us to remove a large quantity of tissue. In addition, we utilized the rectus abdominis fascia or other muscles that are usually exposed during an abdominoplasty. Even in this case, the extension of the exposed muscle, also in the case of reduced undermining of a miniabdominoplasty, consented to the obtaining of enough autologous material to treat all the chosen areas. In the literature, the utilization of the temporal muscular fascia for the facial lifting as well as the grafts from the superficial musculoaponeurotic system (S.M.A.S.) for the lip and nasal-labial folds augmentation is wellknown [2–4].

**Figure 1:**
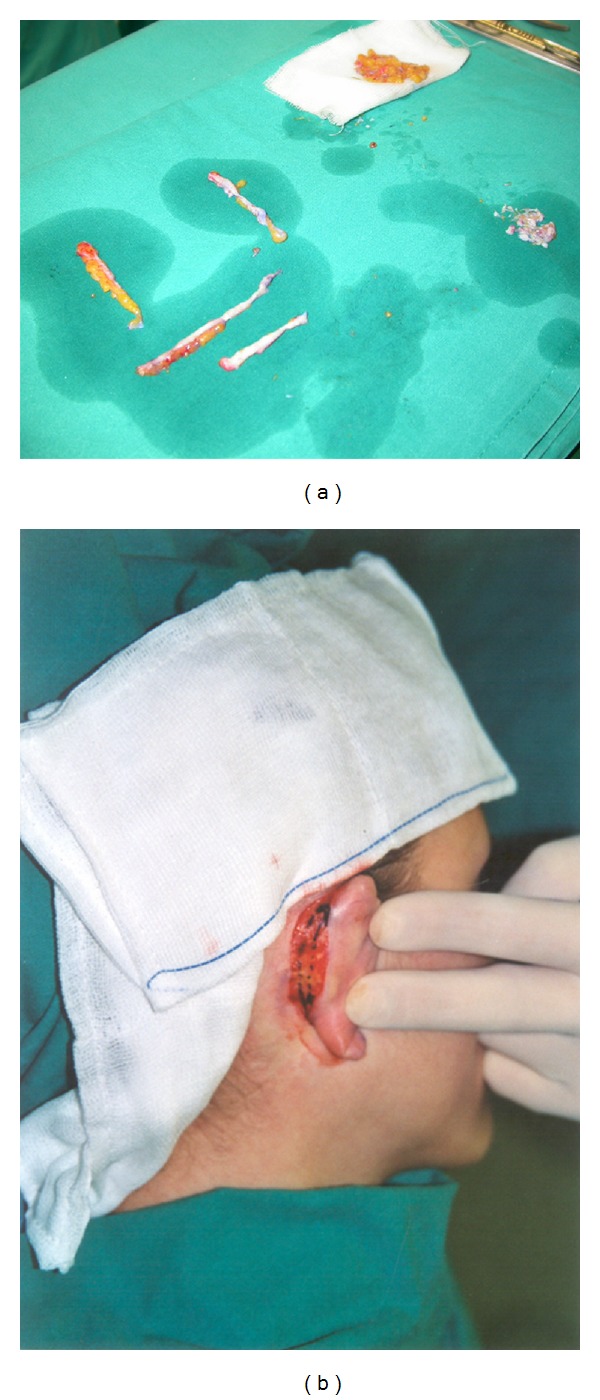
(a) Graft prepared and ready to be inserted during a tight lift, (b) available and utilizable tissue during an otoplasty operation.

 Instead, in the case of operations where the exposition of the muscular plane is not foreseen, we utilized the tissue which more closely offered the characteristics of compactness and adequate thickness suitable for the graft. During an arm lifting or a thighlifting, there was a very compact and easily moulded tissue available in the deeper subcutaneous layer which provides a satisfactory result (Figure 1(a)).

**Figure 2:**
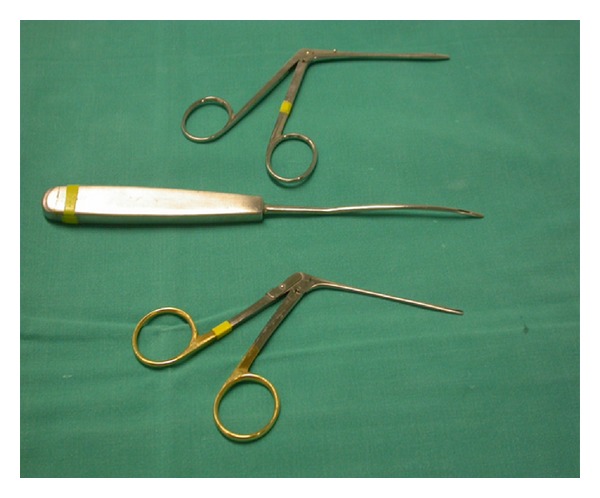
Technique for the insertion of the graft at the intern of the mucous membrane of the labium.

In the case of otoplasty operations, we were also able to obtain tissue with the required characteristics. In this case after removing the skin, we removed and utilized the underlying tissue, even bilaterally, which presented a proper thickness and firmness and which is usually eliminated to make room for the replaced concha auriculae (Figure 1(b)). In order to place the removed tissue, after moulding it, we used a needle which, after entering through a very small incision at one end of the area involved, was passed and came out at the other end. The graft was then fastened to the needle by means of a nylon thread and was replaced internally withdrawing the needle. However, other methods can be easily utilized (Figure 2). In the case of a thin muscular fascia, it is also possible to superimpose two pieces of it and insert them together. The insertion plane is subcutaneous at the level of the nasal-labial folds and is submucosa in the lips. The point of entry as well as that of exit of the needle was closed either by using the cutaneous glue Dermabond or by a stitch in catgut in the case of an incision of the labial mucous membrane. Finally, in the area of removal, it is only necessary to coagulate the exposed underlying muscular fibres or to stitch the two margins of the fascia together.

## 3. Results

**Figure 3:**
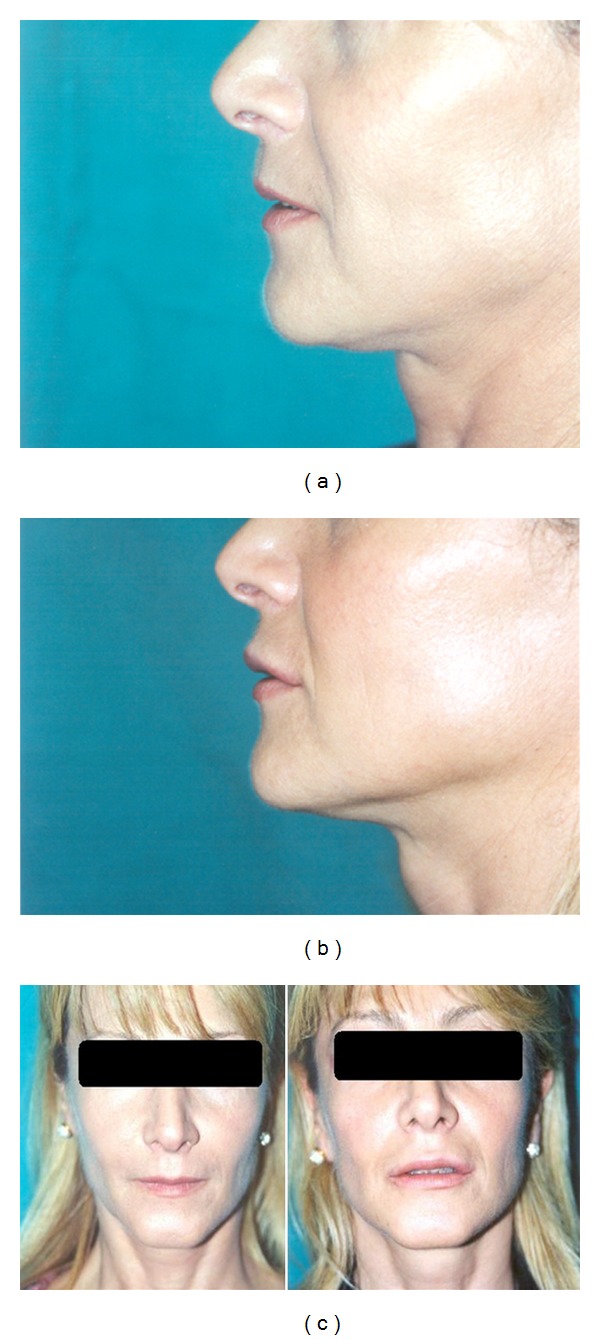
(a, b) are pre- and postoperative results after 20 months. (c) is pre- and postoperative result after 20 months in frontal view.

The results of the treatment of 30 patients were extremely satisfactory (Table 1). The time needed for the insertion was only a few minutes and therefore did not interfere with the total duration of the main operation. Normally using local anaesthesia and sedation in our operations, before the graft, we proceeded with a local anaesthesia of the area to be treated, evidently trying not to alter either the contour or the thickness in order not to compromise the final result. A modest edema lasted for about 5–7 days after the operation. It is also interesting to note that even after more than 20 months from the first graft, we did not find any resorption, and the result obtained appeared to be extremely stable, and such that no initial hypercorrection was carried out (Figures 3(a)–3(c)). 

## 4. Discussion

The possibility of utilizing autologous material for the treatment of the perioral area and that of the lips in order to obtain an aesthetic improvement is certainly advantageous in respect to all other methods. In fact, using this material, there is no rejection whatsoever, there is no foreign-body reactions, and moreover, there is no risk of allergic reactions [5]. It integrates perfectly with the surrounding tissue, guaranteeing a duration in time as well as the naturalness of the result obtained. The evident disadvantages are the necessity of obtaining this material as well as the possible complications during its removal [6]. Since our patients frequently request that these areas be treated at the same time as other operations were undergone, it seemed natural to us to take advantage of the possibility of the removal of tissue wellsuited for the graft during other types of operations.

## 5. Conclusions

With a minimum of imagination, it is possible to discern that in all kinds of operations there is tissue available to be utilized for this purpose without compromising the final result of the main operation.

## Figures and Tables

**Table 1:** Synoptical table concerning a case series of interventions of tissues augmentation.

Patient	Age	Sex	Surgical procedure	Grafting material used	Followup	Complications or revisions
F.T.	38	F	LA	SRAL	20	None
G.F.	42	F	NLFL	PMF	26	None
A.V.	36	F	LA/NLFL	RAF	30	None
R.V.	46	F	NLFL	TDSL	26	None
V.S.	41	F	LA	PMF	28	None
I.M.	44	F	NLFL	PMF	31	None
F.G.	33	F	LA	SRAL	29	None
L.C.	36	F	LA	PMF	29	None
V.R.	44	F	LA/NLFL	RAF	29	None
F.D.G.	51	F	NLFL	TDSL	29	None
L.A.	39	F	LA/NLFL	PMF	30	None
A.M.	42	M	LA	PMF	36	None
G.T.	56	F	NLFL	RAF	29	None
A.P.	52	F	NLFL	TDSL	34	None
B.V.	31	F	LA	SRAL	25	None
M.S.	45	F	NLFL	PMF	26	None
D.V.	47	F	NLFL	PMF	30	None
R.M.	55	M	NLFL	RAF	36	None
E.D.M.	52	F	LA	RAF	36	None
R.B.	51	F	NLFL	TDSL	27	None
P.O.	44	F	NLFL	PMF	31	None
A.L.M.	42	F	LA	PMF	36	None
R.S.	59	F	NLFL	RAF	32	None
R.D.F.	52	F	NLFL	ADSL	31	None
A.C.	50	F	NLFL	PMF	28	None
R.P.	53	F	NLFL	RAF	27	None
S.P.	46	F	NLFL	PMF	33	None
M.G.	51	F	NLFL	ADSL	39	None
T.C.	38	F	LA	PMF	40	None
A.T.	40	F	LA/NLFL	RAF	27	None

*Surgical procedure: *(i) lips augmentation (LA); (ii) nasolabial folds lifting (NLFL).

*Grafting material used: *(i) pectoral muscular fascia (PMF); (ii) rectus abdominis fascia (RAF); (iii) deeper subcutaneous layer from arm (ADSL); (iv) deeper subcutaneous layer from thigh (TDSL); (v) subcutaneous retroauricular layer (SRAL).
